# SR4GN: A Species Recognition Software Tool for Gene Normalization

**DOI:** 10.1371/journal.pone.0038460

**Published:** 2012-06-05

**Authors:** Chih-Hsuan Wei, Hung-Yu Kao, Zhiyong Lu

**Affiliations:** 1 National Center for Biotechnology Information (NCBI), National Library of Medicine, Bethesda, Maryland, United States of America; 2 Department of Computer Science and Information Engineering, National Cheng Kung University, Taiwan, Republic of China; Leuven University, Belgium

## Abstract

As suggested in recent studies, species recognition and disambiguation is one of the most critical and challenging steps in many downstream text-mining applications such as the gene normalization task and protein-protein interaction extraction. We report SR4GN: an open source tool for species recognition and disambiguation in biomedical text. In addition to the species detection function in existing tools, SR4GN is optimized for the Gene Normalization task. As such it is developed to link detected species with corresponding gene mentions in a document. SR4GN achieves 85.42% in accuracy and compares favorably to the other state-of-the-art techniques in benchmark experiments. Finally, SR4GN is implemented as a standalone software tool, thus making it convenient and robust for use in many text-mining applications. SR4GN can be downloaded at: http://www.ncbi.nlm.nih.gov/CBBresearch/Lu/downloads/SR4GN

## Introduction

Species recognition has become increasingly important for the text mining community in recent years. In particular, it has been shown that accurately recognizing species and linking them to relevant genes or proteins is critical to the success of many downstream tasks such as gene normalization (GN) [1], [2] and protein-protein interaction extraction [3], [4], [5]. To address this issue, Gerner et al., [6] developed Linnaeus: a species name identification tool for the biomedical literature. As a standalone and public tool, Linnaeus has been widely used by many participating teams in the BioCreative III Gene Normalization task [1]. More recently, a new hybrid rule-based/machine learning system called OganismTagger [3] was developed as a plugin of the GATE system [7], [8], which was shown to perform favorably to Linnaeus in species identification.

Although identifying species names in biomedical text is not particularly challenging by itself (∼95% in F-measure reported by both Linnaeus and OganismTagger), associating recognized species mentions to other biological entities (e.g. genes) remains challenging and unsolved despite few recent attempts, most notably by Wang et al., [9] and Mu et al., [10].

Based on the pre-annotated gene mentions from the BioCreative I and II GN data [2], [11], Wang and colleagues [9] derived a new corpus named DECA consisting of 644 PubMed citations where in each citation every gene mention was hand tagged with a species ID. Using this corpus, the authors developed a combination method of syntactic parsing and supervised learning, and reported its best performance of 83.80% in accuracy. Their software is made available as a UIMA component of an integrated NLP system [12]. More recently, a hierarchical classification system was developed and experimented with by Mu et al. [10] on the same corpus. A slightly better performance (85.13%) was reported but no software was made available. Unlike their machine-learning based methods, we developed SR4GN: an unsupervised approach that adds new features to our winning system [13] in the 2010 BioCreative III GN task. More specifically, we address two important issues that were not well studied in the past: a) how to determine focus species [14], [15] and b) how to infer species when no explicit organism information can be found in a document (common for abstracts). With a set of new heuristics, SR4GN achieves state-of-the-art performance in benchmarking experiments. The other main contribution of SR4GN lies in its implementation. Inspired by the success of Linnaeus, SR4GN was developed as a standalone, command-line tool that can be readily used to recognize species’ names, map them to NCBI Taxonomy IDs, and associate them with relevant gene/proteins in the biomedical text. With a single download, SR4GN complements and combines the service provided by Linnaeus and Wang et al., [9].

**Figure 1:**
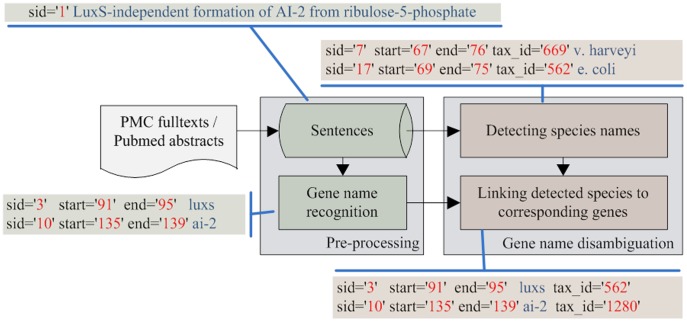
An overview of the SR4GN workflow. Sid, start, end and tax_id in shaded boxes refer to individual sentence identifier; beginning and end text span of a gene or species mention; and NCBI Taxonomy ID.

GNAT [16], [17] is also related to our work in that it attempts to handle multiple species for the GN task. When it was first reported earlier in 2008, GNAT was developed and tested on a benchmark set also derived from the BioCreative I and II GN data. However, its benchmark set contains only a small part of the BioCreative data: 100 abstracts covering 320 genes from 13 species. In addition, GNAT was developed as an integrated approach for GN other than a separate module focusing on identifying species and assigning them to gene mentions. For these two aforementioned reasons, neither GNAT nor its benchmark set was used for comparison in this work.

## Methods

We show in Figure 1 an overview of our SR4GN system. Given as input an abstract or full-length article in either XML or free-text format, both sentence boundaries and gene mentions are first recognized in the preprocessing step. As shown in Figure 1, each sentence is assigned with a sentence identifier (SID). Then by default, we use AIIA-GMT [18] for gene mention recognition but other tools may also be used. Next, SR4GN detects organism names from sentences and assigns them to pre-tagged gene names through the disambiguation step.

### For Species Identification, SR4GN Largely Re-Uses Our Previously Developed Module [13], Which is Primarily a Dictionary Lookup Approach Based on Four Different Resources

The NCBI Taxonomy, handcrafted Linnaeus species dictionary [6], list of species specific cell names (e.g. HeLa cells) from the Cell Line Knowledge Base and Wikipedia. All names and synonyms of a given species from different sources were first normalized to lowercase letters and then automatically transformed into a regular expression for quick lookup (e.g. NCBI Taxonomy ID: 83333 for E. Coli K-12). Whenever possible, our program identifies most specific species. For instance, when a general organism term (e.g. Arabidopsis) co-occurs with a specific name (Arabidopsis thaliana), we would make an inference and associate the NCBI Taxonomy id of the latter also to the former mention. http://clkb.ncibi.org/index.php.

### In Addition to Using Species Names, Cell Names were Found Useful in Species Identification (e.g. HeLa Cells would Indicate Humans). In Particular, We Make a Modification to the Previous System and Created a New Rule in SR4GN as follows

**R1:** When using cell names for inferring species, we relax the requirement in the previous system that the word ‘cell(s)’ appear immediately after a cell name. Instead, we now allow the word ‘cell(s)’ to co-occur in the same noun phrase with a cell name (e.g. HeLa cancer cell).

Note that different from the two general-purpose species recognition tools–Linnaeus and OganismTagger–we also optimized our species recognition module in SR4GN specifically for the gene normalization task. Most notably, we first removed from our dictionary any species that is not linked to any records in Entrez Gene such as Vibrio cholera MO10 (NCBI Taxonomy ID: 345072). Second, we added few common species terms that are absent from the formal terminologies such as “porcine” for Sus scrofa (NCBI Taxonomy ID: 9823). As a result, the number of species for consideration in SR4GN (6,704) is significantly smaller than that in Linnaeus (398,037). Not only does such a reduction in species name space help improve accuracy, but it increases SR4GN’s efficiency too.

### With Respect to Assigning an Identified Taxonomy Id to a Pre-Tagged Gene Mention, Three Heuristic Rules Proposed from the Previous Studies [13], [15] are First Applied in Order

- **Prefix.** If the first lowercase letter of a gene name is an abbreviation of an organism name (e.g. hRrp46p), we assign that species to the gene.
- **Co-occurring word.** If a species and gene name co-occur in the same sentence, we assign that species to the gene. When there are multiple species mentions, priority is given to species mentions appearing to the left of the gene name rather than to its right. When there are multiple species mentions on one side, priority is given to the species name closest to the gene name.
- **Focus species.** We define the most discussed species in a document as the focus species of the document and assign that species to all gene mentions that are not covered by the previous rules. For instance, about 50% of the gene mentions in the DECA corpus [9] need to be assigned by this rule. When two or more species are found with the same number of appearances in a document, we randomly select one as the focus species.

**Table 1:** Evaluation on species detection using the Linnaeus corpus from.

	Precision	Recall	F-measure
SR4GN	86%	85%	86%
Linnaeus	98%	94%	96%
OrganismTagger	96%	63%	76%

### Next, in SR4GN we Added Three New Rules for Addressing the Issues of Focus Species (R2, R3) and Empty Species (R4), Respectively

**R2:** When determining the focus species, we weight the species mention more when it occurs in the title rather than in the abstract. Specifically, we assign double frequency counts to the species mentions in the title when counting their occurrences in a document.

**R3:** Instead of randomly selecting a species when multiple species have the same number of occurrences in a document, we developed a new tie-breaking strategy that uses the global frequency of different species in the Linnaeus corpus as opposed to the random selection in the previous system. For instance, the three most popular species in the Linnaeus corpus are human, rat, and mouse.

**R4:** When no species names can be identified from a document (17% in the DECA corpus), we apply the Species Represented Indicator (SRI) coefficient method [14], which infers four specific species (i.e. human, mouse, yeast, and fly) using words that are strongly correlated with species (e.g. “cohort” for humans and “ferment” for yeast) with a high accuracy of 85%.

**Table 2:** Evaluation on species assignment using the DECA corpus from Wang et al., (2010).

Method	Accuracy
Kao and Wei, 2011	81.08%
+R1	81.61%
+R1+R2	82.10%
+R1+R2+R3	84.20%
**+R1+R2+R3+R4 (SR4GN)**	**85.42%**
Wang et. al., 2010	83.80%
Mu et. al., 2010	85.13%

As in Wang et al., [9] and Mu et al., [10], hand-tagged gene mentions are used.

**Table 3:** Evaluation using the test data from the BioCreative III GN task.

Species Module	TAP-5	TAP-10	TAP-20	F-measure
Kao and Wei, 2010	0.3254	0.3538	0.3535	0.4553
**SR4GN**	**0.3278**	**0.3543**	**0.3543**	**0.4691**
Linnaeus	0.3042	0.3283	0.3283	0.4476
OrganismTagger	0.2915	0.3011	0.3011	0.4456

Both traditional F-measure and BC III TAP-k measure [22] are reported. The same software AIIA-GMT was used to tag gene mentions here. The last two rows show decreased GN results when replacing SR4GN with Linnaeus and OganismTagger for species recognition while keeping all other GN modules (e.g. gene recognition) intact.

**Table 4:** Comparison of benchmarking time on species detection by Linnaeus, OrganismTagger and SR4GN.

System	Loadingdictionary	10 abstracts	100 abstracts	Output format
Linnaeus	41s	1.95s	2.15s	Tab delimited text
OrganismTagger*	34s	37s	5m21s	XML
SR4GN	0	15s	2m44s	XML

SR4GN does not preload the species dictionary into the memory, thus requiring the least amount of computer RAM for the tests shown above: Linnaeus (1.2GB), OrganismTagger (1.6GB), and SR4GN (150MB).

* According to its online documentation, when running 5 parallel threads with 10GB RAM, OganismTagger needs only 14 seconds for processing 100 documents.

**Table 5:** Breakdown of errors by different rules.

Species assignment rules	Applicable genes (%)	# of errors	Accuracy
Prefix	147(2.46%)	0	100%
Co-occurring word	1951(32.66%)	382	80.42%
Focus species	2881(48.23%)	332	88.48%
SRI coefficient method	995(16.66%)	157	84.22%
Total	5974(100%)	871	85.42%

## Results

We conducted various kinds of assessments for SR4GN with respect to its uses in the GN task and compared its performance to that of Linnaeus (v.1.5) and OrganismTagger (v.1.4) whenever possible. First, we evaluated SR4GN’s ability to automatically detect species names from free text using the Linnaeus corpus [6]. As shown in Table 1, SR4GN achieved a precision of 0.86, recall of 0.85 and F-measure of 0.86. Note that in the Linnaeus corpus, every species mention is annotated regardless of their relation to genes. As such, many non-gene linking species were ignored by SR4GN (false negatives). On the other hand, SR4GN makes use of many species cue words like *HeLa Cells*. Although they are found useful for inferring species, they are not annotated in the gold standard (false positives). A third major cause of discrepancies between our computed results and the gold standard can be attributed to the missing and incorrect annotations in the Linnaeus corpus, which was found also in the study by Naderi and colleagues [19].

Secondly, we evaluated SR4GN with respect to its ability to associate detected species to pre-tagged gene mentions. Using the DECA corpus from Wang et al., [9], we show in Table 2 that all of our new rules can help improve system performance. The proposed SR4GN system achieves a significantly better accuracy (85.42%) than our previous system (81.08%) [13]. As shown in Table 2 (last two rows), SR4GN also compares favorably to the two previously reported methods.

Lastly, we have found that using SR4GN can also remarkably improve the results of downstream GN task. As shown in Table 3, using SR4GN while keeping all other parts of our previous BioCreative III GN system unchanged, we can achieve an improved F-score of 0.4691, which would be the best performance on the BioCreative III GN test data ever reported. Also from Table 3, we can see that replacing our SR4GN with the two general-purpose species identification programs–Linnaeus and OrganismTagger–for species identification results in significantly lower GN results (p<0.05 by Fisher’s randomization tests [20]). This suggests that our optimization procedures (e.g. removal of species with no genes in Entrez Gene) are playing a positive and critical role in the GN task.

Finally, we compared SR4GN with Linnaeus and OrganismTagger in terms of computational efficiency (running speed) and output data format, as both are important factors for consideration in practical use besides accuracy. As shown in Table 4, it takes on average 1.5 seconds for SR4GN to process a PubMed abstract on a typical modern desktop computer (with 3.16 GHz CPU and 4 GB RAM), placing SR4GN in between the two existing software. With high-performance and parallel computing, all three tools can be adapted for large-scale document analysis. For instance, SR4GN has been successfully used in batch processing when applied to the entire set of PubMed [21] and the open access subset of PMC.

## Discussion

Despite its solid performance on the DECA corpus, SR4GN failed to assign correct species for approximately 15% of total gene mentions. Hence we analyzed the number of error assignments by each of the heuristic rules in the order as they were applied. As shown in Table 4, the first “prefix” rule achieves perfect precision but it was only applicable to a very small percentage of the total gene mentions.

Compared to other rules, our “Co-occurring word” rule is the least precise when applied to approximately 1/3 of the gene mentions, suggesting that in many cases the co-occurring gene and specie mentions in the same sentence are not straightly correlated. For instance, considering the following sentence (from PMID: 11700027): “Promoter analysis revealed that the intergenic region between Dlad and Uox has promoter activity for both the Dlad and Uox directions, however, the corresponding human genomic fragment has promoter activity only for DLAD.” All gene mentions (e.g. Dlad) in the first part of the sentence are annotated as mouse genes based on the entire abstract despite that they are co-located with the species mention “human” in this sentence.

As for the “focus species” rule, SR4GN decides the focus species to be the one that is the most discussed in the text. As shown in Table 5, this rule is applicable to nearly half of the gene mentions. But unfortunately, the rule is not always correct. On the DECA corpus, 64.2% (213/332) of the errors were due to the fact that the focus species was not the most mentioned species. This rule also fails when the focus species is completely missing from the examined text (focus species appears only in the full text but not abstract), which accounted for the remaining 35.8% of the errors.

Lastly, due to confusion and ambiguity in the species indicating words between human and mouse, the SRI coefficient method fails to distinguish these two species in many occasions. Indeed, about 80% of SRI errors (125/157) made an incorrect assignment between these two species.

## Conclusion

Here we report SR4GN, a standalone system for recognizing species mentions and pairing them with corresponding gene/protein mentions. Unlike existing general-purpose species recognition tools, SR4GN is optimized for the gene normalization task. By incorporating new rules for the specific problems of identifying focus species and inferring species when no explicit species mention can be found in a document, SR4GN performs better than the previous systems when benchmarked on public data sets. In addition, we believe SR4GN is computationally efficient to handle large-scale text mining applications. Our error analysis suggests modifying the co-occurring rule and applying SR4GN to full text (when available) may result in future enhancement of SR4GN’s performance. Other future investigation includes additional evaluation beyond the species data in the current DECA corpus.

## Availability

http://www.ncbi.nlm.nih.gov/CBBresearch/Lu/downloads/SR4GN.
